# 78 Classification and Regression Tree Model for Predicting Satisfaction with Life Scale Scores After Burn Injury

**DOI:** 10.1093/jbcr/irac012.081

**Published:** 2022-03-23

**Authors:** Zephorah Bissoon, Emma L Gause, Gretchen J Carrougher, Claudia Baker, Shelley A Wiechman, Tam N Pham, Nicole S Gibran, Barclay T Stewart

**Affiliations:** Johns Hopkins University, Baltimore, Maryland; Harborview Medical Center, Seattle, Washington; Department of Surgery, The University of Washington, Seattle, Washington; Harborview Medical Center, Seattle, Washington; University of Washington, Seattle, Washington; University of Washington, Seattle, Washington; University of Washington, Wellfleet, Massachusetts; University of Washington, Seattle, Washington

## Abstract

**Introduction:**

Current early burn care prognostication models predict in-hospital mortality (e.g., revised Baux Score). However, patients, families and clinicians need more holistic tools in the hours and days after injury to identify specific factors that might affect their quality of life and indicate a need for more intensive services. This project aims to predict Satisfaction with Life (SWL) in survivors of burn injury using patient, injury, and care factors available within 24 hours of admission.

**Methods:**

Two hundred and fourteen participants were identified from a multicenter national longitudinal database and merged with clinical data from a single institution's trauma registry. Patients were randomized into a training dataset (80%) and a testing dataset (20%). A CART algorithm was used to examine the relative contributions of individual predictor variables in classifying low SWL at six-month follow up (SWL ≤ 20). Seventeen covariables obtained within 24 hours of index hospital admission were analyzed from five domains: demographics, comorbidities, injury, care, and host response to injury. Lab values were those closest to but not greater than 24 hours after index hospital admission.

**Results:**

Multiple covariables contributed to the SWL score. CART analysis selected a pre-injury SWL score < 31 as the first node and strongest indicator of low SWL. CART then selected the following subgroups at risk for SWL ≤ 20 at 6 months: (1) hematocrit >55%; (2) lactate >4 mmol/L, age > 59; (3) total body surface area (TBSA) burned >30%, presence of a hand, neck, and/or face burn. The cross-validated predictive accuracy of the CART model was 69.4% with a cross-validated relative error of 0.379. In the validation data set, sensitivity and specificity were 62.5% and 72.0%, respectively.

**Conclusions:**

The findings demonstrate the potential feasibility of creating a model that can predict a clinically meaningful quality of life outcome using covariables gathered within hours of hospital admission after burn injury. Predictive measures suggest that while some of the included covariables may be associated with SWL, they are not consistently and reliably predictive of low SWL alone. With more data and additional refined inputs, a similar model could be used to identify those in need of more intensive services earlier on in the hospitalization.

